# Prevalence of venous thromboembolism and evaluation of a modified caprini risk assessment model: a single-centre, prospective cohort study involving patients undergoing lung resections for bronchiectasis

**DOI:** 10.1186/s12959-022-00402-1

**Published:** 2022-08-01

**Authors:** Qingshan Chen, YongSheng Cai, Zhirong Zhang, Honghong Dong, Jinbai Miao, Hui Li, Bin Hu

**Affiliations:** Department of Thoracic Surgery, Beijing Institute of Respiratory Medicine and Beijing Chao-Yang Hospital, Capital Medical University, Beijing, China

**Keywords:** Bronchiectasis, Surgery, Venousthromboembolism, Caprini risk assessment modle

## Abstract

**Background:**

Venous thromboembolism (VTE) is a common postoperative complication in general thoracic surgery, but the incidence of patients undergoing surgery for bronchiectasis was not known. The purpose of our study was to investigate the incidence of VTE in bronchiectasis patients undergoing lung resection and to evaluate the risk stratification effect of the modified caprini risk assessment model (RAM).

**Methods:**

We prospectively enrolled patients with bronchiectasis who underwent lung resection surgery between July 2016 and July 2020.The postoperative duplex lower-extremity ultrasonography or(and) computed tomographic pulmonary angiography (CTPA) was performed to detect VTE. The clinical characteristics and caprini scores of VTE patients and non-VTE patients would be compared and analyzed. Univariate logistic regression was performed to evaluate whether higher Caprini scores were associated with postoperative VTE risk.In addition, We explored the optimal cutoff for caprini score in patients with bronchiectasis by using the receiver operating characteristic (ROC) curve.

**Results:**

One hundred and seventeen patients were eligible based on the prospective study criteria. The postoperative VTE incidence was 8.5% (10/117). By comparing the clinical characteristics and Caprini scores of VTE and non-VTE patients, the median preoperative hospitalization (7 vs 5 days, *P* = 0.028) and Caprini score (6.5 vs 3,*P* < 0.001) were significantly higher in VTE patients. In univariate logistic regression, a higher Caprini score was associated with higher odds ratio (OR) for VTE of 1.7, 95% confidence interval (CI) was from 1.2 to 2.5 (*P* = 0.001), C-statistics was 0.815 in the modified caprini RAM for predicting VTE. In a multivariable analysis adjusting for preoperative hospitalization, a higher Caprini score was associated with higher odds OR for VTE of 1.8 (95%CI: 1.2–2.6, *P* = 0.002), C-statistics was 0.893 in the caprini RAM for predicting VTE. When taking the Caprini score as 5 points as the diagnostic threshold, the Youden index is the largest.

**Conclusions:**

The postoperative VTE incidence in patients undergoing lung resection for bronchiectasis was 8.5%.The modified caprini RAM effectively stratified bronchiectasis surgery patients for risk of VTE and showed excellent predictive power for VTE. The patients with postoperative caprini scores = 5, should be recommended to take positive measures to prevent postoperative VTE.

**Trial Registration:**

Chinese Clinical Trial Register: ChiCTR-EOC-17010577.

## Introduction

Bronchiectasis, a complex multicomponent disease, is associated with a vicious cycle of abnormal airway inflammation, chronic bacteria colonization and recurrent infection with bronchial structural irreversible damage [1]. The growing prevalence of bronchiectasis in both developing and developed countries has been recognized over the past decades [2–4], augmenting the demand for operation intervention.

Venous thromboembolism(VTE) usually refers to deep venous thrombosis(DVT) and pulmonary embolism(PE). In 1856, Virchow proposed three precipitants for venous thrombosis: venous stasis or immobilization; hypercoagulability;and vascular damage [5]. Numerous researches have suggested that various factors could alter the Virchow’s triad and increase the risk of VTE. Acute or chronic infection could affect venous stasis or increase coagulability of the blood [6–8], surgical procedures could not only directly damage blood vessels but also affect endothelial function [6]. Inflammation, the responses of the immune system to infection and injury, is a key determinant of endothelial function and promotes activation of the coagulation system [9]. In addition, the BTS guidelines [10] point out that some connective tissue diseases may be the cause of bronchiectasis, including antiphospholipid syndrome, primary Sjögren's syndrome, and systemic lupus erythematosus. Therefore, there may be an inherited or acquired hypercoagulable state preoperatively [11].Thus, there have been several mechanisms increasing the risk of postoperative VTE in bronchiectasis surgery patients. Previous studies showed that postoperative VTE was associated with increased complications and short-term mortality risk [12–15].

In addition to infection and inflammation, surgery also increases the risk of VTE.Patients undergoing bronchiectasis surgery, especially thoracotomy, may be susceptible to VTE. Although video-assisted thoracoscopic surgery (VATS) is safe and effective in lung resection, there are still many patients with bronchiectasis who have to choose thoracotomy due to severe lung tissue adhesion, and the rate of conversion to open thoracotomy(OT) is as high as 15.3% [16]. Furthermore,a recent research result show that a more hypercoagulable post-operative state in patients who underwent OT than in those who underwent VATS [17]. Therefore, it is necessary to focus on the occurrence of postoperative VTE in patients with bronchiectasis undergoing lung resection. However, the incidence of postoperative VTE in bronchiectasis patients was still not known.

More recently, to reduce the incidence of postoperative VTE, the Caprini risk assessment model (RAM), an individual VTE risk assessment model, has been widely used in the field of surgery. Multiple specialties, including plastic and reconstructive, general, gynecologic surgery and thoracic oncology surgery, have evaluated the caprini RAM, finding that the caprini RAM could effectively risk-stratify patients and identify patients at high risk for postoperative VTE [18–23]. Subsequently, application of a modified caprini RAM in thoracic surgery showed better predictive power, with low risk (0–4 points), intermediate risk (5–8 points), and high-risk (= 9 points) [20]. A prospective study about VTE after lung resection in China also demonstrated this [24]. However,it is unclear whether its risk stratification matches the risk level of VTE after surgery in patients with bronchiectasis.The predictive power of the modified caprini RAM on risk-stratifying patients undergoing lung resection for bronchiectasis has not been evaluated and validated.

Therefore, we aimed to determine the incidence of postoperative VTE in bronchiectasis surgery patients, and to evaluatethe power of modified caprini RAM to identify patients at high risk for VTE after lung resection in patients with bronchiectasis.

## Patients and methods

### Study design and population

From July 2016 to July 2020, patients who underwent lung surgery for bronchiectasis were included in the study. The exclusion criteria included: (i). non-localized bronchiectasis; (ii). due to any reason, preoperative cardiopulmonary function tests was not completed; (iii).due to any reason, preoperative lower extremity vascular ultrasound was not completed. (iv). preoperative DVT by duplex lower-extremity ultrasonography examinations; (v).any patients who had to be anticoagulated perioperatively were excluded; (vi). after surgery before discharge, duplex lower-extremity ultrasonography examinations were not completed.CTPA was necessary to detect PE in patients with newly diagnosed DVT postoperatively, or with typical symptoms of PE (chest pain, dyspnea or persistent hypoxemia), or Caprini score = 9. Duplex lower-extremity ultrasonography examinations was performed according to the appointment time, usually within one week after the patient's lung resection. The endpoint was confirmed VTE or patient discharge.

All patients’ medical records were reviewed, including age, gender, body mass index (BMI), hemoptysis, special infection (tuberculosis, fungus, or pseudomonas aeruginosa), surgical procedure (thoracotomy or minimally invasive surgery), the extent of resected lobes, operation time, the volume of blood loss, and length of preoperative hospitalization.

### Postoperative VTE and caprini score

VTE events were diagnosed and identified by using duplex lower-extremity ultrasonography or CTPA. The primary goal of our study was to prospectively investigate the incidence of postoperative VTE in bronchiectasis surgery patients without perioperative chemoprophylactic anticoagulation.The secondary goal was to evaluate the risk-stratification and predictive power of modified caprini RAM for VTE.The caprini scores were calculated and summarized postoperatively based on the modified caprini RAM [18, 25].

### Statistical analysis

Statistical comparison between non-VTE patients and VTE patients was accomplished using the ?^2^ test or Fisher’s exact test for categorical variables, 2-sample t-tests or the Wilcoxon rank sum test for continuous variables. VTE incidence assessment and distribution were calculated based on the Caprini score. Univariate logistic regression was performed to evaluate whether higher Caprini scores were associated with postoperative VTE risk. The significant confounders in baseline characteristics were included in the multivariate logistic regression. The Hosmer–Lemeshow test and C-statistic were used to assess the goodness of fit and test discrimination for logistic regression models. To explore the appropriate positive test cutoff value for VTE, we generated and analyzed the ROC with the Caprini score as the predictor and the postoperative VTE event as the response. Sensitivity, specificity, and 95%CI, accuracy, and positive and negative predictive values were calculated for all thresholds. R, version 4.0.2 (R Foundation for Statistical Computing, Vienna, Austria) was used to build and analyze the ROC. All statistical analyses were performed using SAS V9.4 (SAS Institute, Cary, NC, USA). A value of *p* < 0.05 was considered statistically significant.

## Results

### Incidence of postoperative VTE

**Fig. 1:**
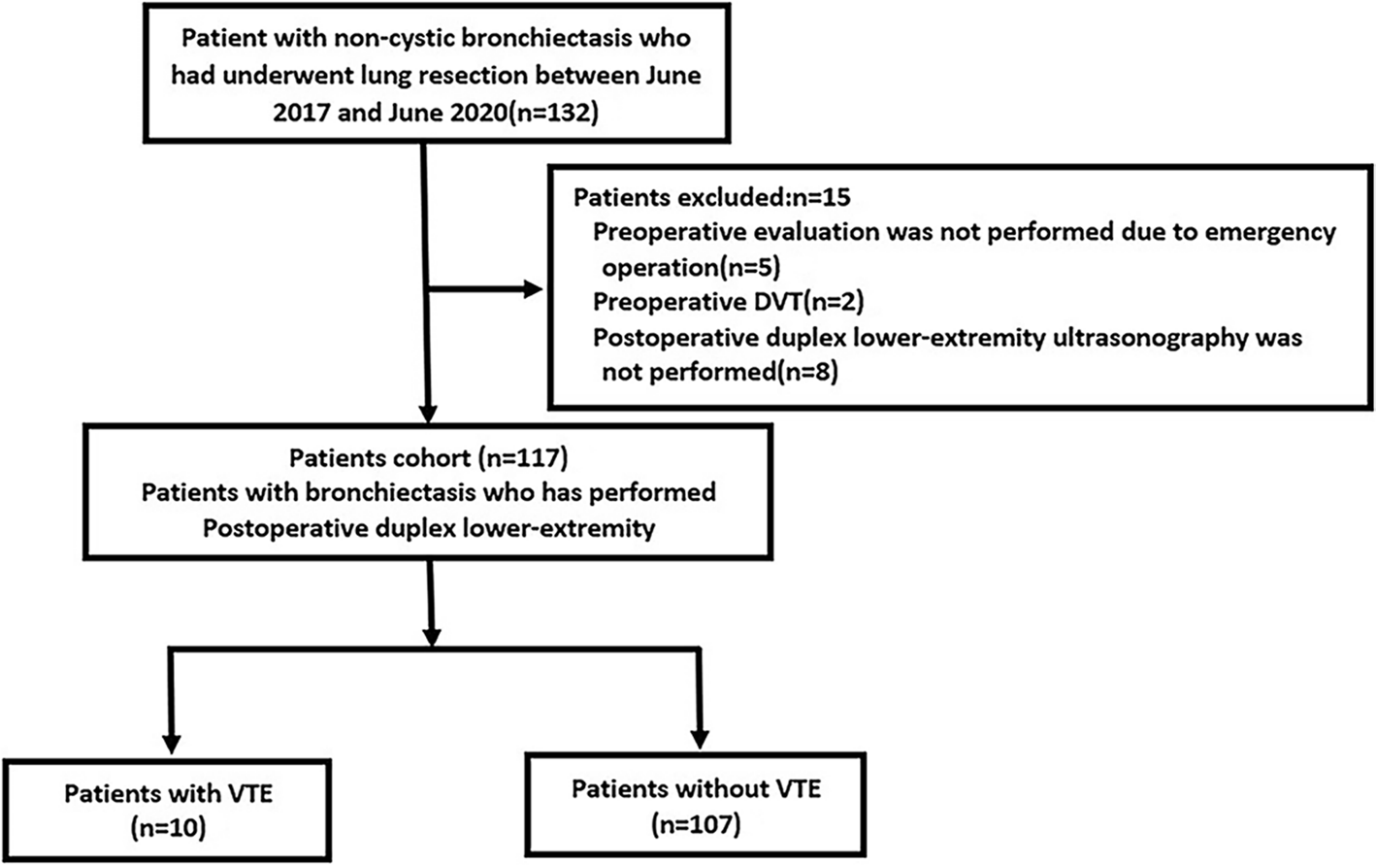
Selection of the study cohort. DVT, deep venous thrombosis; VTE, venous thromboembolism

From July 2016 to July 2020, 132 patients underwent surgery for bronchiectasis. Five of these patients underwent emergency surgery without routine preoperative assessment due to massive hemoptysis; DVT was found in two patients who received therapeutic anticoagulation before surgery; postoperative duplex lower-extremity ultrasonography examination was missed in eight patients because the appointment of duplex ultrasound was later than the time of discharge from the hospital. The remaining 117 patients were eventually eligible for the prospective study criteria. The overall incidence of postoperative VTE comprised 8.5% (10/117) during hospitalization (Fig. 1).

Of all VTE events: eight were only DVTs, two were DVTs and PEs.In DVT events,there were five patients with proximal and distal lower extremity thrombus, respectively (Table 1). Among them, five patients with distal lower extremity DVT were all gastrocnemius venous thrombosis. There was one patient with superficial femoral vein and profunda femoral vein, respectively. Both femoral and popliteal veins thrombosis occurred in two patients. Thrombosis in superficial femoral, profunda femoral and common femoral veins in one patient. One of the PEs affected the left pulmonary artery, and the other showed segmental pulmonary arteries of the left upper lobe, but neither had typical clinical manifestation of PE (such as serious chest pain, dyspnea or hypoxemia). All VTE events, except one, were diagnosed within 1 week after surgery. The median time from surgery to VTE assessment was 3 days, interquartile range (IQR) was 2.And median time from surgery to VTE events was 4.5 days, interquartile range (IQR) was 4 (Table 1).

**Table 1:** Postoperative venous thromboembolism events

Patients	Surgical procedures	VTE location	Time from Surgery to VTE
1	VATS Lobectomy	DVT: gastrocnemius vein	5 days
2	Thoracotomy Lobectomy + segmentectomy	DVT: superficial femoral vein	5 days
3	VATS Lobectomy	DVT: profunda femoral vein PE: segmental pulmonary arteries of the left upper lobe	2 days
4	VATS Lobectomy	DVT: gastrocnemius vein	4 days
5	VATS Lobectomy + segmentectomy	DVT: femoral and popliteal veins	6 days
6	VATS Lobectomy	DVT: gastrocnemius vein	9 days
7	Thoracotomy Lobectomy	DVT: superficial femoral, profunda femoral, common femoral veins PE: left pulmonary artery	6 days
8	VATS Lobectomy	DVT: gastrocnemius vein	1 day
9	VATS Lobectomy + Wedge resection	DVT: gastrocnemius vein	2 days
10	Thoracotomy Pneumonectomy	DVT: femoral and popliteal veins	4 days

*VTE* venous thromboembolism, *VATS* video-assisted thoracoscopic surgery, *DVT* deep venous thrombosis, *PE* pulmonary embolism

### Baseline characteristics by VTE

The median length of preoperative hospitalization in the VTE group was 7 days (IQR,5), significantly longer than that in the non-VTE group (median,5; IQR, 3) (*P* = 0.028). There was no significant difference among gender, BMI, hemoptysis, special infection, surgical procedures, the extent of resected lobes, operation time, and the volume of blood loss between the VTE group and non-VTE group (Table 2).

**Table 2:** Baseline characteristics of Non-VTE and VTE groups

	Non-VTE (107)	VTE (10)	P
Male^a^	46(43)	3(30)	0.645
Age(years)^b^	51.4(12.5)	57.9(5.4)	0.130
BMI(kg/m^2^)^b^	23.4(3.1)	23.5(3.2)	0.969
Symptom^a^			1.000
Hemoptysis	69(64.5)	6(60)	
No-Hemoptysis	38(35.5)	4(40)	
Special infection^a,d^	27(25.2)	5(50)	0.093
Tuberculosis^a^	0	1(10)	0.086
Fungus^a^	25(23.3)	4(40)	0.434
Pseudomonas aeruginosa^a^	4(3.7)	1(10)	0.366
Procedure^a^			1.000
VATS	75(70)	7(70)	
Thoracotomy	32(30)	3(30)	
Resected Lobes^a^			0.634
= 1 lobe	72(67.3)	6(60)	
1–2 lobes	28(26.2)	3(30)	
> 2 lobes	7(6.5)	1(10)	
Operation time(min)^b^	161.7(56.7)	171(58.2)	0.620
Lose blood(ml)^b^	312.4(548.6)	153(78)	0.589
preoperative hospitalization(day)^c^	5(3)	7(5)	0.028

*BMI* body mass index, *SD* standard deviation, *IQR* interquartile range

^a^n(%)

^b^mean (SD)

^c^median (IQR).VTE, venous thromboembolism; VATS, video-assisted thoracoscopic surgery

^d^Special infection: including tuberculosis, fungus, and Pseudomonas aeruginosa

### Postoperative caprini score

Risk factors and Caprini scores are summarized in Table 3. Median Caprini scores (median, 6.5; IQR, 4) in the VTE group were significantly different from those in the non-VTE group (median, 3; IQR, 1) (*P* < 0.001).

**Table 3:** Caprini risk factors and scores by VTE events

Caprini risk factors	weighted scores	Non-VTE	VTE	*P*
Age 40–59 years	1	58(54.2%)	2(20%)	0.082
Abnormal pulmonary function	1	13(12.2%)	3(30%)	0.276
BMI = 30 kg/m^2^	1	2(1.9%)	0(0%)	1.000
Congestive heart failure (< 1 month),	1	2(1.9%)	2(20%)	0.036
History of inflammatory bowel disease	1	0(0%)	1(10%)	0.086
Sepsis (< 1 month)	1	13(12.2%)	4(40%)	0.055
Serious acute lung disease (< 1 month)	1	3(2.8%)	3(30%)	0.008
Varicose vein	1	2(1.9%)	0(0%)	1.000
Age 60–74 years	2	28(26.2)	8(80%)	0.002
Central venous access	2	3(2.8%)	1(10%)	0.304
Confined to bed (> 72 h)	2	4(3.7%)	2(20%)	0.082
Major open surgery (= 45 min)	2	107(100%)	10(100%)	1.000
prior cancer (except nonmelanoma skin)	2	7(6.5%)	0(0%)	1.000
Age = 75 years	3	1(0.9%)	0(0%)	1.000
History VTE	3	6(5.6%)	1(10%)	0.474
Chemotherapy	3	1(0.9%)	0(0%)	1.000
Caprini score, median (IQR)	-	3(1)	6.5(4)	< 0.001

Values are n (%) unless otherwise indicated

*VTE* venous thromboembolism, *BMI* body mass index, *IQR* interquartile range

**Fig. 2:**
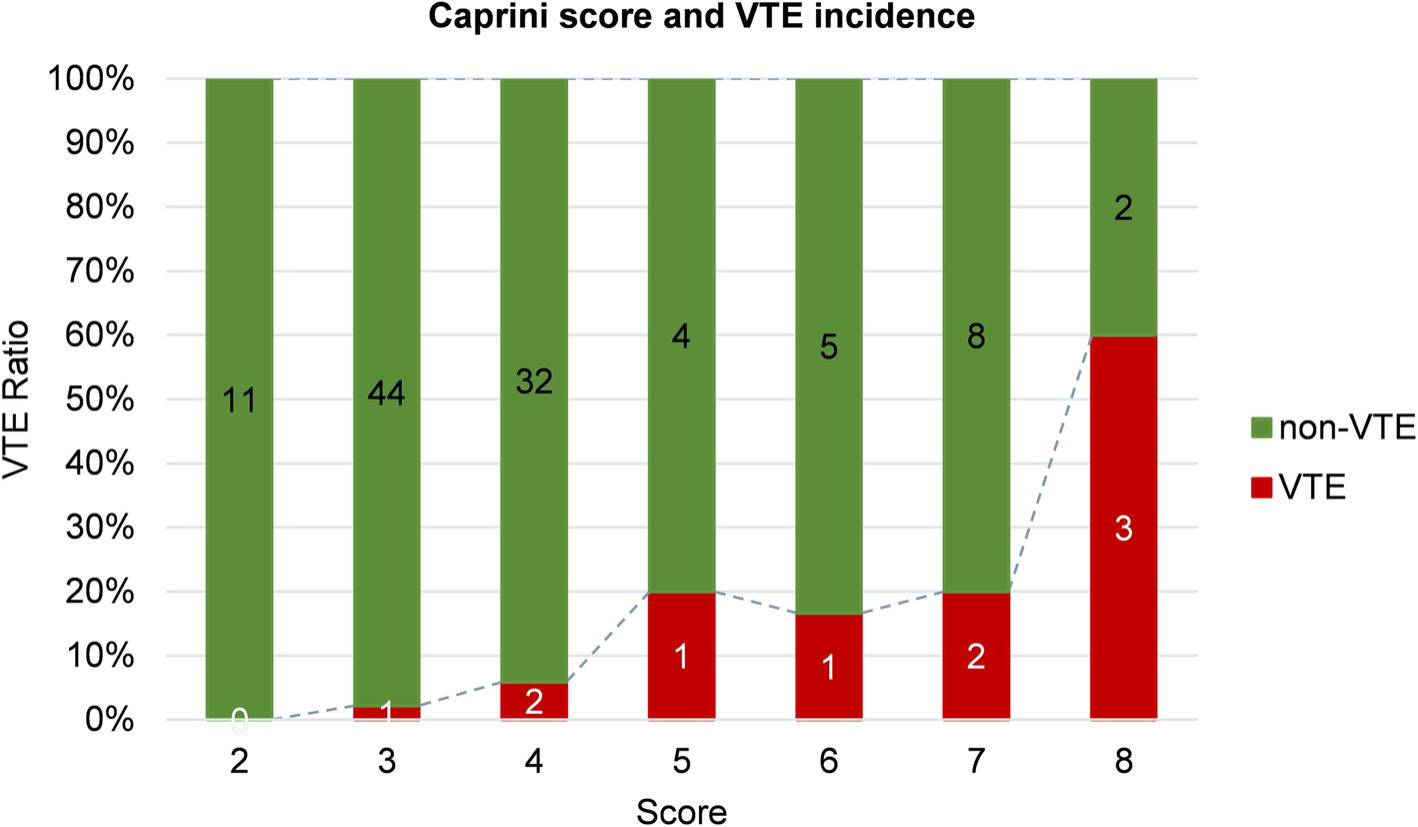
VTE distribution and incidence based on Caprini score. Caprini scores are displayed on the y-axis, bars representing VTE and non-VTE ratio are plotted on x-the axis. The number in the bars represents the frequencies of patients. VTE, venous thromboembolism

Except for one patient with a Caprini score = 9, the Caprini score was < 9 in all patients.The distribution of VTE and non-VTE events based on caprini scores were shown in Fig. 2. From Caprini scores 2 to 8, the VTE incidence was 0, 2.2% (1/45), 5.9% (2/34), 20% (1/5), 16.7% (1/6), 20% (2/10), and 60% (3/5), respectively.

### Caprini model assessment

On univariate logistic regression analysis, a higher Caprini score conferred a 1.7 OR of postoperative VTE (95%CI: 1.2–2.5, *P* = 0.001). After adjusting for length of preoperative hospitalization, a higher Caprini score was still associated with an increased OR of VTE of 1.8 (95%CI: 1.2–2.6, P = 0.002). C-statistics for the association between Caprini score and the probability of VTE were 0.815 and 0.893, respectively. Hosmer–Lemeshow goodness-of-fit for both univariate and multivariate logistic regression models were satisfied (Table 4).

**Table 4:** Univariate and multivariate logistic regression models for Caprini RAM versus VTE probability

Measure	Caprini Score VS VTE	
	Univariate *P*	Multivariate *P*
Caprini Score, OR (95%CI)	1.7(1.2–2.5) 0.001	1.8(1.2–2.6) 0.002
Preoperative hospitalizations OR (95%CI)	-	1.3(1.1–1.7) 0.035
C-statistic	0.815	0.893
Hosmer–Lemeshow		
?^2^/df	0.69/3	2.44/8
* P* Value	0.876	0.964

*OR (95%CI)* odds rations(95% confidence intervals), *VTE* venous thromboembolism, *RAM* risk assessment model, *df* degrees of freedom

**Fig. 3:**
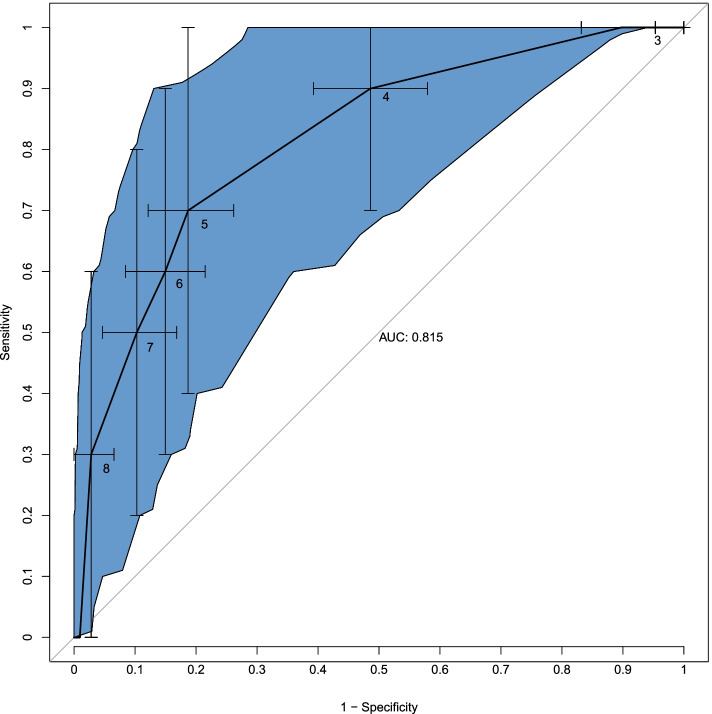
ROC curve with Caprini score as the predictor and VTE event as the response. The blue area represents 95% confidence intervals for sensitivity and “1 – specificity.” Points are labeled with corresponding Caprini score thresholds. For thresholds 2 to 8, sensitivities were 100%, 100%, 90%, 70%, 60%, 50%, 30%, respectively; “1 – specificities” were 100%, 89.7%, 48.6%, 19.7%, 15%, 10.3%, 2.8%, respectively

The ROC with Caprini score as the predictor and postoperative VTE events as the response, with 95% CIs for sensitivity and “1 – specificity” was shown in Fig. 3. Based on the Youden index, we choose the optimal positive test cutoff of 5 points.With a Caprini score of 5 set as the positive test cutoff, the resultant specificity was 81.3% (95%CI: 73.8%-87.9%), sensitivity was 70.0% (95%CI: 40.0%-100%), accuracy was 80.3%, and the negative and positive predictive values were 73.1% and 78.9%, respectively.

## Discussion

The current prospective study demonstrated that the incidence of postoperative VTE after lung resection for bronchiectasis was still substantial. 8.5% patients had a postoperative VTE. In addition, we evaluated the capacity of Caprini RAM to stratify bronchiectasis surgery patients for VTE risk. A higher Caprini score was associated with a greater likelihood of postoperative VTE events. A Caprini score of 5, used as a positive test cutoff, seemed to be most appropriate based on excellent sensitivity and specificity. Although there have been many studies on VTE after oncological lung resection and esophagectomy, few data exist on the incidence of postoperative VTE in patients undergoing surgical treatment for bronchiectasis. Only several reports have shown the incidence of postoperative VTE in patients with benign lung disease. For instance, previous studies at our center [24, 26]reported that the incidence of postoperative VTE was 7% in patients with benign lung disease. However, notably, due to the limited sample size of only 27 patients with bronchiectasis, this study did not determine a more reliable incidence of postoperative VTE in patients with bronchiectasis. Likewise, Ala-Seppälä et al. [27]analyzed the occurrence of VTE in more than 400 patients undergoing surgical intervention for pleural infections during long-term follow-up and found that the incidence of VTE was 3.8% at three months, 5.0% at one year, 8.8% at three years, and 12.4% at five years. However, the definite number of pleural infections caused by bronchiectasis is unknown. Again, the incidence of postoperative VTE in patients undergoing surgery for bronchiectasis remains unclear. In the present study, we recorded the high incidence of postoperative VTE in patients undergoing surgical procedures for bronchiectasis.

In this study, the risk of postoperative VTE in patients with bronchiectasis would increase as the caprini score increased, demonstrating the modified caprini RAM is helpful in predicting postoperative VTE. However, the risk stratification of this system may not be perfect for patients with bronchiectasis.In clinical practice, patients tended to be classified into different risk categories based on the postoperative caprini score to assist in VTE prevention. In thoracic surgery, the modified caprini scoring system (low: 0–4, moderate: 5–8; high: = 9) was widely used, which could not only stratify the risk of postoperative VTE in patients with lung and esophageal cancer, but also guide postoperative VTE prophylaxis [20, 28–30]. Current guideline suggested patients at low risk for VTE should receive mechanical prophylaxis without chemoprophylactic anticoagulation due to a low incidence of postoperative VTE (< 1.5%) [31]. However, there were some drawbacks in the application of the modified Caprini scoring system to bronchiectasis patients. In this study, only 1 patient had a postoperative Caprini score = 9, when the modified Caprini scoring system was applied to patients with bronchiectasis, almost all the patients were considered as low to moderate risk category postoperatively, and it was not able to distinguish the patients at truly high risk for VTE. Moreover, the risk stratification of the modified Caprini scoring system did not match the risk level of postoperative VTE in patients with bronchiectasis.76.9% (90/117) of patients with Caprini score = 4 was at low risk for VTE, the VTE incidence in the low risk category was 3.3% (3/90), nearly twice as high as 1.5% [31], suggesting that patients with Caprini score = 4 included some truly moderate to high risk patients, and receiving mechanical prophylaxis alone may not be sufficient. Further researches were needed on the appropriate Caprini scoring system for bronchiectasis patients.

The excellent predictive effect of the Caprini RAM also contributed to the diagnosis of postoperative VTE in bronchiectasis. Previous study showed that the best positive test cutoff value for VTE in lung cancer surgery patients was 9, the resultant sensitivity, specificity, accuracy, positive and negative predictive value were 83.3%, 60.5%, 61.6%, 10.3% and 98.5%, respectively [20]. For esophagectomy patients, the best positive test cutoff value was 15, the resultant sensitivity, specificity, accuracy, positive and negative predictive value were 100%, 66.7%, 71.4%, 33.3% and 100%, respectively [28]. The patients (Caprini score = the best positive test cutoff value) may benefit from enhanced prophylactic anticoagulation [20, 28]. However, unlike lung and esophageal tumor patients, the postoperative Caprini scores of almost all patients undergoing surgery for bronchiectasis were lower than 9 due to much more younger patients and absence of tumor risk factors [20, 28]. According to the ROC with Caprini scores as postoperative VTE predictor, in bronchiectasis patients undergoing lung resection, a Caprini score of 5 was set as a positive test cutoff with excellent sensitivity and specificity, high positive predictive value and negative predictive value. These patients (Caprini score = 5) should be recommended for positive VTE monitoring and prevention postoperatively. Therefore, there should be individual positive test cut-off values for different kinds of diseases to assist in postoperative VTE prevention in thoracic surgery.

There were some limitations to our study. First, our study was a single-center study and had a small sample size, thus the conclusion may be biased. Additionally, we had no prolonged follow-up for patients after discharge. The VTE risks among patients undergoing lung resection for bronchiectasis may have been underestimated in this cohort. Larger sample sizes and long-term follow-up studies were needed.

## Conclusions

In conclusion, the overall postoperative VTE incidence in patients undergoing lung resection for bronchiectasis was still substantial. Integration of Caprini RAM had the potential to risk-stratify patients, especially when the postoperative Caprini scores were equal or more than 5, these patients should be recommended to enhance screen for postoperative VTE and receive positive prophylaxis program.

## Data Availability

The datasets used in the current study are available from the corresponding author on reasonable request.
